# The *Caenorhabditis elegans* DEG-3/DES-2 Channel Is a Betaine-Gated Receptor Insensitive to Monepantel

**DOI:** 10.3390/molecules27010312

**Published:** 2022-01-05

**Authors:** Tina V. A. Hansen, Heinz Sager, Céline E. Toutain, Elise Courtot, Cédric Neveu, Claude L. Charvet

**Affiliations:** 1 INRAE, Université de Tours, ISP, F-37380 Nouzilly, France; tvalstrup@icloud.com (T.V.A.H.); elise.courtot@inrae.fr (E.C.); cedric.neveu@inrae.fr (C.N.); 2 Elanco Animal Health Inc., CH-4058 Basel, Switzerland; heinz.sager@elancoah.com; 3 Elanco Animal Health Inc., F-92317 Sèvres, France; celine.toutain@elancoah.com

**Keywords:** nAChR, betaine, *Caenorhabditis elegans*, *Xenopus laevis*, DEG-3/DES-2, monepantel

## Abstract

Natural plant compounds, such as betaine, are described to have nematocidal properties. Betaine also acts as a neurotransmitter in the free-living model nematode *Caenorhabditis elegans*, where it is required for normal motility. Worm motility is mediated by nicotinic acetylcholine receptors (nAChRs), including subunits from the nematode-specific DEG-3 group. Not all types of nAChRs in this group are associated with motility, and one of these is the DEG-3/DES-2 channel from *C. elegans*, which is involved in nociception and possibly chemotaxis. Interestingly, the activity of DEG-3/DES-2 channel from the parasitic nematode of ruminants, *Haemonchus contortus*, is modulated by monepantel and its sulfone metabolite, which belong to the amino-acetonitrile derivative anthelmintic drug class. Here, our aim was to advance the pharmacological knowledge of the DEG-3/DES-2 channel from *C. elegans* by functionally expressing the DEG-3/DES-2 channel in *Xenopus laevis* oocytes and using two-electrode voltage-clamp electrophysiology. We found that the DEG-3/DES-2 channel was more sensitive to betaine than ACh and choline, but insensitive to monepantel and monepantel sulfone when used as direct agonists and as allosteric modulators in co-application with betaine. These findings provide important insight into the pharmacology of DEG-3/DES-2 from *C. elegans* and highlight the pharmacological differences between non-parasitic and parasitic nematode species.

## 1. Introduction

Parasitic nematodes of crops, livestock, companion animals and humans have long been a major burden as they cause losses of food, morbidity and mortality in animals and humans. The estimated economic loss worldwide due to nematodes in the agricultural sector is almost 125 billion $ annually [1], whereas the combined annual cost of parasitic helminth infections in ruminants is estimated to 1.8 billion € in Europe alone [2].

Before the advent of the synthetic anthelmintics, natural compounds were important to control parasitic nematodes. Nowadays, such compounds, from both terrestrial plants and marine algae (seaweeds), are becoming more popular because of the increasing spread of anthelmintic resistance and the negative impact of synthetic nematocidal compounds on the biodiversity [3,4,5]. Several marine algae have been explored as alternative treatment options against parasitic nematodes. One of these is the brown alga, *Ascophyllum nodosum*, which extract is rich in betaine (i.e., γ-aminobutyric acid betaine, δ-aminovaleric acid betaine and glycine betaine), and which alkaline extracts have shown to arrest larval development and suppress the fecundity of the tomato root-knot nematodes *Meloidogyne javanica* and *M. incognita* [6,7,8]. Betaine is a ubiquitous non-canonical amino acid that acts as an osmolyte, as a methyl donor and has anti-inflammatory effects in mammals [9]. In the free-living model nematode *Caenorhabditis elegans*, betaine acts as a neurotransmitter, and is required for a basal level of motility shown to be mediated by the ACR-23 nicotinic acetylcholine choline receptor (nAChR) [10]. These nAChRs are pentameric proteins that are either homomeric- or heteromeric ligand-gated ion channels of the Cys-loop family, and are expressed in neuronal-, non-neuronal and muscle cell membranes [11,12]. *Caenorhabditis elegans* possesses one of the most extensive and diverse nAChR-subunit gene families [13], separated into five groups based on sequence homology and named after the first of their number to be discovered [14]. Among these groups, the nematode specific-DEG-3 group [13], contains 8 genes, including the founding members *deg-3* and *des-2*, which constitute the DEG-3/DES-2 channel, preferentially gated by choline as compared to acetylcholine when expressed in *Xenopus laevis* oocytes [15,16]. The DEG-3/DES-2 channel has been localized to several types of neurons within *C. elegans*, such as the PVD neurons, the PVC neurons, and likely the sensory endings of the sensory neurons FLP, and the chemosensory neurons IL-2 in the head region [15,16]. Due to the localization of the DEG-3/DES-2 channel in sensory endings of chemosensory neurons, it was shown to function in sensory neurons [17] and proposed to be involved in chemosensation [15]. More recently, *acr-20* and *acr-23*, which are other members of the DEG-3 group, were shown to encode functional homomeric channels preferentially activated by betaine compared with choline [10,18]. However, the effect of betaine on nematode DEG-3/DES-2 channels has never been investigated so far. From an anthelmintic point of view, it is interesting that several ion channels, constituted of subunits from the DEG-3 group, are activated not only by betaine, but also by the amino-acetonitrile derivatives (AADs), a recent chemical class of anthelmintics (i.e., monepantel and monepantel sulfone) [19]. Monepantel sulfone, the major metabolite of monepantel in sheep [20], allosterically activates the DEG-3/DES-2 channel from the parasitic nematode of ruminants *Haemonchus contortus* [21]. Monepantel and its sulfone metabolite also act as direct agonists on the DEG-3 group members: *Cel*-ACR-23 channel [22], the *Cel*-ACR-20 channel and its homologue from *H. contortus*, the MPTL-1 channel [18]. However, the action of AADs on *Cel*-DEG-3/DES-2 has not yet been assayed.

Since betaine, monepantel and monepantel sulfone are described to activate several nAChRs formed by subunits from the DEG-3 group, we hypothesized that betaine, monepantel and/or monepantel sulfone could activate or potentiate the *Cel*-DEG-3/DES-2 channels. Here we aim at advancing the pharmacological knowledge of the DEG-3/DES-2 channel from *C. elegans*. Our results show that the *Cel*-DEG-3/DES-2 channel is highly sensitive to betaine, but insensitive to monepantel and monepantel sulfone both as agonists and allosteric modulators. Therefore, these findings highlight a different pharmacology of the DEG-3/DES-2 channel between parasitic and non-parasitic nematodes.

## 2. Results

### 2.1. The C. elegans DEG-3/DES-2 Channel Is a Betaine-Sensitive Receptor

**Figure 1:**
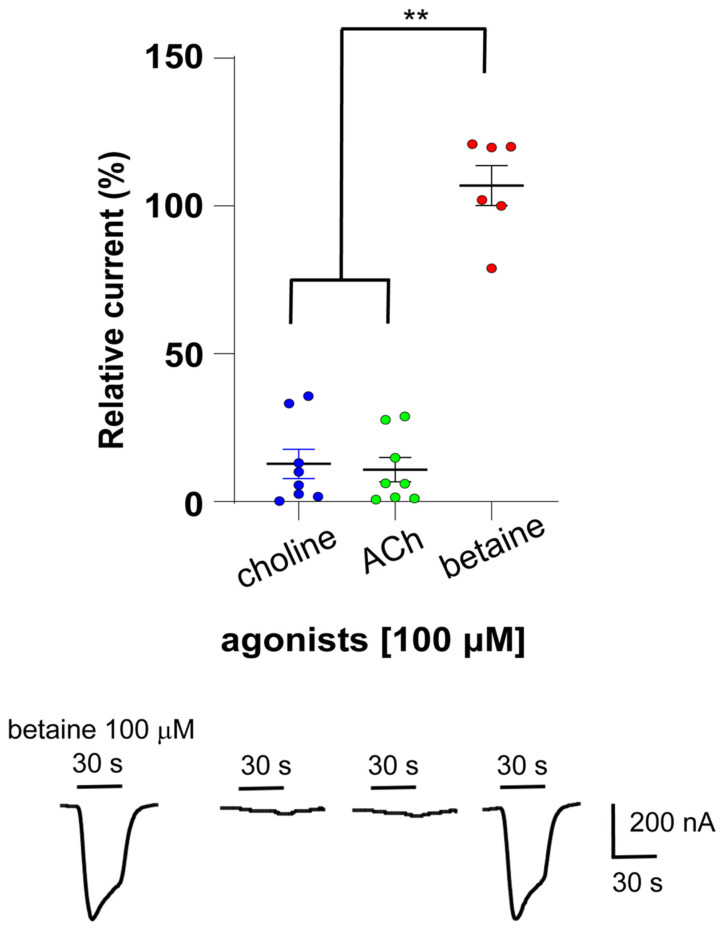
Effect of agonists on the *Cel*-DEG-3/DES-2 channel. A scatter dot plot and representative traces show the rank order efficacy in nA of ACh, choline and betaine. Current responses are normalized to 100 µM betaine-elicited currents and given as mean ± SEM (*n* = 6–8), ** *p* < 0.002. The perfusion time of each compound was 30 s as indicated with short bars above the traces.

The *Cel*-DEG-3/DES-2 channel was previously described to be preferentially activated by choline relatively to acetylcholine [15] while other members from the DEG-3 group such as *Cel*-ACR-20 and *Cel*-ACR-23 gave rise to receptors more sensitive to betaine than choline. In order to investigate the effect of betaine on *Cel*-DEG-3/DES-2, we PCR-amplified and cloned the full-length coding sequences of the *Cel-deg-3* and *Cel-des-2*. The amino acid sequences of the cloned subunits were 100% identical to *Cel*-DES-2 and *Cel*-DEG-3 sequences available in GenBank (NM_001392615 and NM_001392614, respectively). Subsequently, we expressed the *Cel*-DEG-3/DES-2 channel in oocytes and assayed acetylcholine (ACh), choline and betaine, all at a concentration of 100 µM using two-electrode voltage-clamp electrophysiology. As expected, 100 µM ACh and choline elicited weak current responses in the nA range, whereas the perfusion of 100 µM betaine led to robust currents at approximately 0.5 μA. The relative current responses of these compounds are given in Figure 1 as a scatter dot plot with normalized means ± SEM, along with representative traces for each agonist. Strikingly, the last betaine-elicited currents were significantly larger than ACh- (*p* = 0.002) and choline-evoked currents (*p* = 0.001), corresponding to 15.7 ± 4.6% and 22.4 ± 7.6% of the initial 100 µM betaine response, respectively. The last betaine response was in the same range 106.9 ± 6.8% as the first one (*p* > 0.9). The rank order potency series for the compounds tested was betaine >>> choline = ACh.

**Figure 2:**
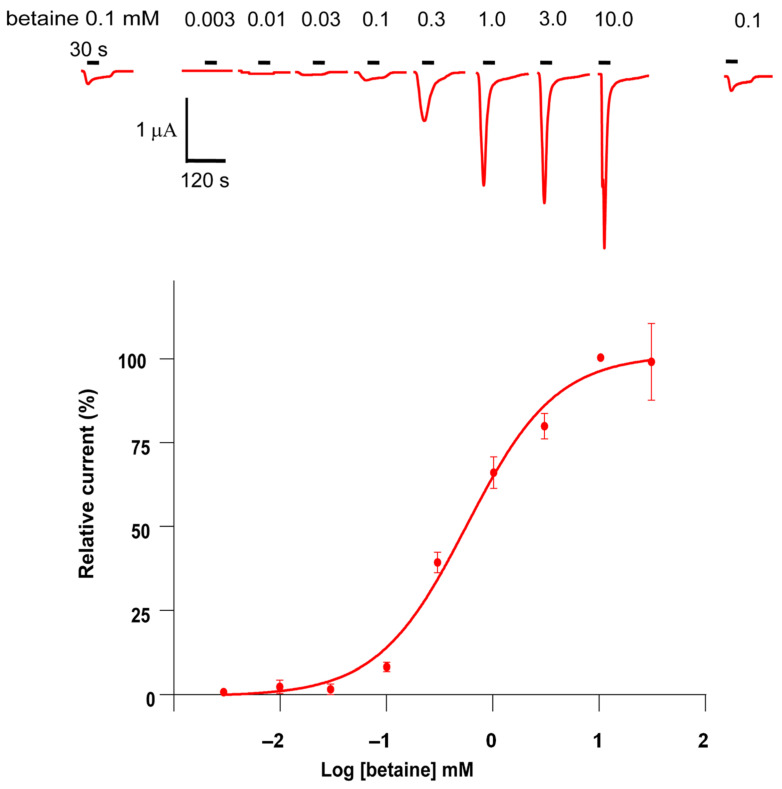
Concentration-response relationship of betaine. Individual oocytes expressing the *Cel*-DEG-3/DES-2 channel were challenged with increasing concentrations of betaine. The number of oocytes (*n*) used were *n* = 32, except for betaine at 30 mM where *n* = 7. All current responses were normalized to current responses induced by 10 mM betaine and given as mean ± SEM with representative traces. The perfusion time of each compound was 30 s as indicated with short bars above the traces.

Then, to characterize the sensitivity of *Cel*-DEG-3/DES-2 for betaine, we established a dose-response relationship by challenging the oocytes with betaine concentrations ranging from 0.003 to 30 mM. Figure 2 shows the concentration-response relationship for betaine on the *Cel*-DEG-3/DES-2 channel obtained from 3 individual experiments (*n* = 7, 15 and 10, respectively). The maximum current amplitudes were obtained with 10 mM betaine and were used to normalize all responses. The betaine concentration-response curve was characterized by an EC_50_ value of 0.6 ± 0.2 mM and a Hill coefficient of 1.0 ± 0.02 (*n* = 32). Thus, we find betaine is a new agonist of the *Cel*-DEG-3/DES-2 channel which has a higher potency than choline and ACh.

### 2.2. The C. elegans DEG-3/DES-2 Channel Is Not Modulated by Monepantel and Monepantel Sulfone

**Figure 3:**
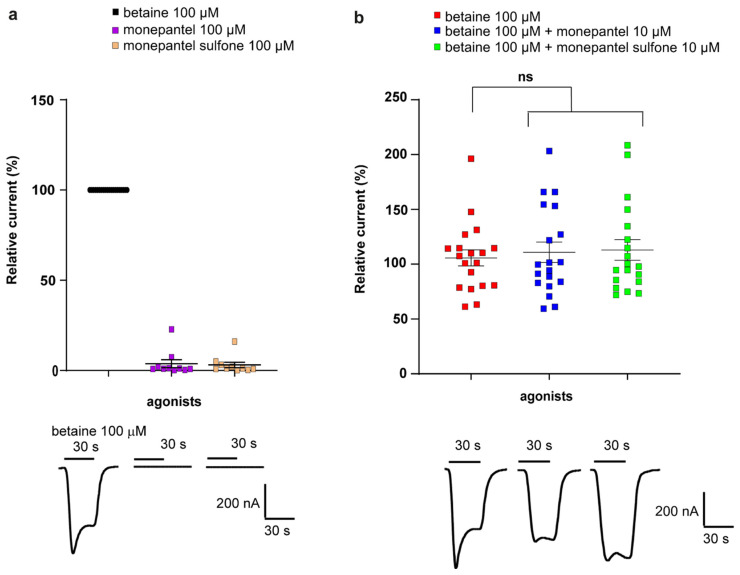
Effect of monepantel and monepantel sulfone on the *Cel*-DEG-3/DES-2 channel. Scatter dot plots (mean ± SEM) and representative traces show the efficacy of monepantel and monepantel sulfone used as direct agonists (*n* = 10) (**a**) and as allosteric modulators in co-application with betaine (*n* = 19) (**b**). The perfusion time was 30 s as indicated with short bars above the traces.

Nematode channels from the DEG-3 group, including *Cel*-ACR-20, *Cel*-ACR-23 and *Hco*-MPTL-1, *Hco*-DEG-3/DES-2, were previously reported to be sensitive to monepantel and monepantel sulfone, either as direct agonists and/or as allosteric modulators [18,21,22]. We therefore tested the response of the *Cel*-DEG-3/DES-2 channel to direct applications of monepantel and monepantel sulfone and co-applications of these compounds with 100 µM betaine. The relative current responses of monepantel and monepantel sulfone and their respective co-application with betaine are given in Figure 3a,b as scatter dot plots with normalized means ± SEM, along with representative traces for each type of application. Monepantel and monepantel sulfone failed to activate the *Cel*-DEG-3/DES-2 channel when applied at 100 µM for 30 s (responses were below 4% of the response to 100 µM betaine) (Figure 3a). Co-application of 100 µM betaine with 10 µM monepantel or 10 µM monepantel sulfone, did not induce a significant higher or lower response as compared to 100 µM betaine alone (*p* = 0.9), but in some experiments we did observe minor changes in the current kinetics. Hence monepantel and monepantel sulfone were neither acting as direct agonists or allosteric modulators on the *Cel*-DEG-3/DES-2 channel (Figure 3b).

## 3. Discussion

It was first described that the co-expression of *C. elegans* DEG-3 and DES-2 subunits resulted in a functional heteromeric acetylcholine-gated channel in *X. laevis* oocytes [16]. Furthermore, Yassin et al. showed that choline had a slightly higher affinity (EC_50_ of 1.8 +/− 0.7 mM) and efficacy (13-fold higher responses) compared to ACh (EC_50_ of 2.9 +/− 0.5 mM) on the DEG-3/DES-2 channel [15]. However, the low affinity of choline and ACh raises the question of those ligands being the only physiological agonists of the DEG-3/DES-2 channel. Since several channels formed by subunits from the DEG-3 group were reported to be sensitive to betaine, we investigated whether the *Cel*-DEG-3/DES-2 channel could also be sensitive to this compound. In the present study, we found that betaine is a novel agonist of the *Cel*-DEG-3/DES-2 channel, with an EC_50_ value of 0.6 ± 0.2 mM, which is 3-fold lower than that of choline. Similarly, betaine was described to be the preferred agonist of the *Cel*-ACR-23 channel (EC_50_ value of 1.4 mM) [10] while choline acted as a partial agonist, unable to saturate the channel [22]. The *Cel*-ACR-20 channel was shown to be more sensitive to betaine (EC_50_ of 25 ± 7 µM) while its choline sensitivity was in the same range as *Cel*-DEG-3/DES-2 (EC_50_ of 1.2 +/− 0.3 mM) [18]. In the livestock nematode parasite *H. contortus*, *mptl-1* is the homologous gene for *Cel-acr-23* and encodes a betaine- (EC_50_ 41 ± 7 µM) and choline- (EC_50_ of 1.3 +/− 0.2 mM) gated channel [18]. Altogether, these data support that betaine responsiveness is the hallmark of channels from the DEG-3 group. Therefore, it is tempting to speculate that the *Hco*-DEG-3/DES-2, characterized by a choline EC_50_ of 9.9 +/− 2.5 mM, would also be preferentially activated by betaine, which requires further experimental studies [21]. The sensitivity to betaine is interesting as the *Cel*-DEG-3/DES-2 channel has been proposed to be needed for the chemotaxic response to choline [15]. Assuming the *Cel*-DEG-3/DES-2 channel also mediates a chemotaxic response to betaine, our results suggest that the *Cel*-DEG-3/DES-2 channel could also be involved in betaine detection. It is possible that the chemotaxic response of the worm (i.e., move towards or away from betaine) is concentration-dependent. Although betaine is a neurotransmitter in *C. elegans* that is required for a basal level of motility, and well tolerated in the environment at 50 mM, betaine inhibits the motility of *C. elegans* at a higher concentration (i.e., 250 mM) [10]. Choline is an essential- and betaine an important micronutrient, at least in humans, and they are interconnected in biosynthesis pathways [23]. Choline is a precursor for both betaine and ACh. Choline is acetylated to the neurotransmitter ACh, or oxidized to betaine which is subsequently used in methylation pathways, or choline is included in the biosynthesis of the phospholipid, phosphatidylcholine (PtdCho), a major structural component of cellular membranes [23]. The chemotaxic response of *C. elegans* induced by choline [15], suggests that this compounds may also be essential in this nematode species. It is interesting that betaine inhibits the motility of *C. elegans* at high concentrations [10], and suppresses fecundity and arrests larval development of the plant parasitic nematodes *M. javanica* and *M. incognita* [6,7,8]. The DEG-3/DES-2 channel of plant parasitic nematodes have, to our knowledge, not yet been functionally characterized and exploited as potential targets for nematocides. Since betaine may be the hallmark of channels from the DEG-3 group, and affects the fecundity and larval development of *Meloidogyne* spp., we speculate whether members of the DEG-3 group, particularly the DEG-3/DES-2 channel, could be inducing the nematocidal effect reported for the tomato root-knot nematodes *Meloidogyne* spp. Further investigations may identify potential nematocidal targets in the DEG-3 group from plant nematodes.

Anthelmintics from the AAD chemical class, such as monepantel and monepantel sulfone, have been reported to act either as direct agonists on the *Cel*-ACR-20, *Cel*-ACR-23 and *Hco*-MPTL-1 channels [18,22] and/or as allosteric modulators on the *Cel*-ACR-20, *Hco*-MPTL-1 and *Hco*-DEG-3/DES-2 channels by strongly enhance the non-desensitization current induced by choline [21]. In our study, we did not observe any allosteric effect during co-application of betaine with monepantel or monepantel sulfone, suggesting that the DEG-3/DES-2 channel does not represent one of the targets for AADs in *C. elegans*. These results are in accordance with Rufener et al. [24], who reported no loss of monepantel sensitivity in the DEG-3/DES-2 *C. elegans* mutant strain [24]. Hence, the *C. elegans* DEG-3/DES-2 channel is pharmacologically distinguishable from the *H. contortus* DEG-3/DES-2 channel. The difference in binding sites that cause the pharmacological distinguishability remains to be elucidated.

## 4. Materials and Methods

### 4.1. Drugs

All drugs were purchased at Sigma-Aldrich (Saint-Quentin Fallavier, France) with the exception of monepantel and monepantel sulfone which were provided by Elanco Animal Health. Stock solutions were prepared in either recording solution (100 mM NaCl, 2.5 mM KCl, 1 mM CaCl_2_, 5 mM HEPES, pH 7.3) or DMSO (100%) and subsequently dissolved in recording solution with a maximum final concentration of 0.1% DMSO.

### 4.2. Cloning of the des-2 and deg-3 Subunits from C. elegans

Total RNA was extracted from the Bristol N2 wild-type strain of *C. elegans*, obtained from the *Caenorhabditis* Genetics Center (CGC), using TRI Reagent (Molecular Research Center, Inc., Cincinnati, OH, USA). First strand cDNA synthesis was performed using 4 μL of total RNA and the Maxima H minus Reverse Transcriptase kit (Thermo Scientific, Waltham, MA, USA) according to the manufacturers’ recommendations. The full-length coding sequences of *Cel-des-2* and *Cel-deg-3* were obtained with nested PCRs using the Phusion High Fidelity Polymerase (New England BioLabs, Ipswich, MA, USA) and the first strand cDNA as a template. Primer sequences were designed based on the coding sequences of the *Cel-des-2* (GenBank accession number NM_001392615), and the *Cel-deg-3* (NM_001392614). The first round of PCRs were performed with the primer-combination F0/R0 and the second round with F1/R1 as described previously [25]. The primer pair sequences were as followed for *Cel-des-2* and *Cel-deg-3*, respectively: F0-*Cel-des-2* 5′-gacaaccccactttttggtcca-3′, R0-*Cel-des-2* 5′-cgcgtcatgtgtgtggggga-3′, F1-*Cel-des-2*-pTB207-*XhoI* 5′-gcggccgctcgagatgcttattattattcaaagcttgc-3′, R1-pTB207-*ApaI* 5′-accagatcaagctcgggccctcatcctccatattcaacaccag-3′ and F0-*Cel-deg-3* 5′-gtccctaaccaaattttacaggt-3′, R0-*Cel-deg-3* 5′-caaagtaccggtacacatcgc-3′, F1-*Cel-deg-3*-pTB207-*XhoI* 5′-gcggccgctcgagatgacgttaaagattcggacaatc-3′, R1-*Cel-deg-3*-pTB207-*ApaI* 5′-accagatcaagctcgggcccttagacattaaagaatcggtcatc-3′. Purified PCR products were cloned into the expression vector pTB207 [26] using the In-Fusion HD Cloning kit (Clontech, Mountain View, CA, USA). Constructs were verified by sequencing (Eurofins Genomics GmBH, Ebersberg, Germany). The pTB207-*Cel-des-2* construct was linearized with the restriction enzymes *PaeI* and *MscI* and the pTB207-*Cel-deg-3* construct with *MscI and PstI* (Thermo Scientific, Waltham, MA, USA). cRNAs were synthesized using the mMessage mMachine T7 transcription kit (Ambion, Austin, TX, USA), purified on NucleoSpin RNA columns (Macherey-Nagel GmbH, Düren, Germany) and kept at −80 °C until use.

### 4.3. Electrophysiological Experiments in Xenopus laevis Oocytes

*Xenopus laevis* oocytes were purchased from Ecocyte Biosciences (Dortmund, Germany) and kept at 19 °C in incubation solution (100 mM NaCl, 2 mM KCl, 1.8 mM CaCl_2_, 1 mM MgCl_2_, 5 mM HEPES, 2.5 mM C_3_H_3_NaO_3_, 100 μg/mL streptomycin and 100 U/mL penicillin, pH 7.3). Each oocyte was co-injected with cRNAs of *Cel-des-2* (0.2 μg/μL) and *Cel-deg-3* (0.2 μg/μL) in a total volume of 36 nL using the Drummond nanoject II microinjector (Drummond Scientific Company, Broomall, PA, USA). After 4–5 days incubation, the function and the pharmacological properties of the receptors were explored on oocytes clamped at −80 mV by two-electrode voltage-clamp performed with the fully-automated system Robocyte2 (Multichannel systems MCS GmbH, Reutlingen, Germany). All experiments were performed with oocytes pre-incubated in 100 μM BAPTA-AM for 3.5 h to chelate intracellular Ca^2+^ ions and prevent activation of endogenous calcium activated chloride channels [26]. All drugs were applied for 30 s, and each drug application separated by a 2 min wash-out period. To test the relative potency of ACh, choline and betaine, each oocyte was initially exposed to 100 μM betaine and subsequently to 100 μM of ACh, choline and betaine. Peak current values were normalized to the first peak current response to 100 μM betaine. The concentration response relationship for betaine was performed by challenging oocytes with increasing concentrations (3 µM–30,000 µM). The peak current values were normalized to the response to 10,000 µM betaine giving the maximum current amplitude. Monepantel and monepantel sulfone were assayed, by applying each oocyte with 100 µM betaine followed by 100 µM monepantel or monepantel sulfone alone to investigate an agonistic effect, while the allosteric modulating effect was evaluated by the co-application of 100 µM betaine with 10 µM monepantel or 10 µM monepantel sulfone. All responses were normalized to the current response obtained with 100 μM betaine. All electrophysiological data was analyzed with Clampfit 10.7 (Molecular Devices, Sunnyvale, CA, USA) and GraphPad Prism 9 (GraphPad Software, La Jolla, CA, USA) on normalized current values. For the drug efficacy tests, the normalized drug-group means were statistically analyzed using a non-parametric Kruskal–Wallis Test with a Dunn’s multiple comparison test, where *p* < 0.05 was considered significant. The dose-response relationship of betaine was established by fitting the normalized currents as a function of drug concentration to a Hill equation using non-linear regression analysis with a variable slope model. The following equation was used:

*I*_rel_ = *I*_min_ + (*I*_max_ − *I*_min_)/(1 + 10^((LogEC50 − [*D*])*n_H_)^)

where *I*_rel_ is the mean relative current, *I*_max_, is the relative current obtained at saturating agonist concentration, *I*_min_ is the relative current obtained at agonist concentrations 0 µM, EC_50_ is the concentration of agonist at which 50% of the maximal current response is obtained, [*D*] is the drug concentration and *n*_H_ is the Hill coefficient. *I*_max,_ EC_50_ and *n*_H_ were fitted as free parameters whereas *I*_min_, was constrained to 0.

## 5. Conclusions

The results described in this study provide important additional insight into the pharmacology of the DEG-3/DES-2 channel from *C. elegans*, highlight the pharmacological difference between a non-parasitic and a parasitic nematode species, and arise questions on the nAChR subtypes of the DEG-3 group in parasites as targets for the development of natural nematocidal compounds.

## Data Availability

Data sharing not applicable.

## Sample Availability

Samples of the compounds are not available from the authors.

## References

[1] Chitwood D.J. (2003). Research on plant-parasitic nematode biology conducted by the United States Department of Agriculture-Agricultural Research Service. Pest Manag. Sci..

[2] Charlier J., Rinaldi L., Musella V., Ploeger H.W., Chartier C., Vineer H.R., Hinney B., von Samson-Himmelstjerna G., Băcescu B., Mickiewicz M. (2020). Initial assessment of the economic burden of major parasitic helminth infections to the ruminant livestock industry in Europe. Prev. Vet. Med..

[3] Ghareeb R.Y., Adss I.A., Bayoumi S.R., El-Habashy D.E. (2019). The nematicidal potentiality of some algal extracts and their role in enhancement the tomato defense genes against root knot-nematodes. Egypt J. Biol. Pest Control.

[4] Rose H., Rinaldi L., Bosco A., Mavrot F., de Waal T., Skuce P., Charlier J., Torgerson P.R., Hertzberg H., Hendrickx G. (2015). Widespread anthelmintic resistance in European farmed ruminants: A systematic review. Vet. Rec..

[5] Prichard R. (1994). Anthelmintic resistance. Vet. Parasitol..

[6] Wu Y., Jenkins T., Blunden G., Whapham C., Hankins S.D. (1997). The role of betaines in alkaline extracts of *Ascophyllum nodosum* in the reduction of *Meloidogyne javanica* and *M. incognita* infestations of tomato plants. Fundam. Appl. Nematol..

[7] Wu Y., Jenkins T., Blunden G., Von Mende N., Hankins S.D. (1998). Suppression of fecundity of the root-knot nematode, *Meloidogyne javanica*, in monoxenic cultures of *Arabidopsis thaliana* treated with an alkaline extract of *Ascophyllum nodosum*. J. Appl. Phycol..

[8] Whapham C.A., Jenkins T., Blunden G., Hankins S.D. (1994). The role of seaweed extracts, *Ascophyllum nodosum*, in the reduction in fecundity of *Meloidogyne javanica*. Fundam. Appl. Nematol..

[9] Zhao G., He F., Wu C., Li P., Li N., Deng J., Zhu G., Ren W. (2018). Betaine in inflammation: Mechanistic Aspects and Applications. Front. Immunol..

[10] Peden A.S., Mac P., Fei Y.J., Castro C., Jiang G., Murfitt K.J., Miska E.A., Griffin J.L., Ganapathy V., Jorgensen E.M. (2013). Betaine acts on a ligand-gated ion channel in the nervous system of the nematode *C. elegans*. Nat. Neurosci..

[11] Albuquerque E.X., Pereira E.F.R., Alkondon M., Rogers S.W. (2009). Mammalian Nicotinic Acetylcholine Receptors: From Structure to Function. Physiol. Rev..

[12] Holden-Dye L., Joyner M., O’Connor V., Walker R.J. (2013). Nicotinic acetylcholine receptors: A comparison of the nAChRs of *Caenorhabditis elegans* and parasitic nematodes. Parasitol. Int..

[13] Jones A.K., Sattelle D.B. (2004). Functional genomics of the nicotinic acetylcholine receptor gene family of the nematode, *Caenorhabditis elegans*. Bioessays.

[14] Brown L.A., Jones A.K., Buckingham S.D., Mee C.J., Sattelle D.B. (2006). Contributions from *Caenorhabditis elegans* functional genetics to antiparasitic drug target identification and validation: Nicotinic acetylcholine receptors, a case study. Int. J. Parasitol..

[15] Yassin L., Gillo B., Kahan T., Halevi S., Eshel M., Treinin M. (2001). Characterization of the DEG-3/DES-2 receptor: A nicotinic acetylcholine receptor that mutates to cause neuronal degeneration. Mol. Cell Neurosci..

[16] Treinin M., Gillo B., Liebman L., Chalfie M. (1998). Two functionally dependent acetylcholine subunits are encoded in a single *Caenorhabditis elegans* operon. Proc. Natl. Acad. Sci. USA.

[17] Cohen E., Chatzigeorgiou M., Husson S.J., Steuer-Costa W., Gottschalk A., Schafer W.R., Treinin M. (2014). *Caenorhabditis elegans* nicotinic acetylcholine receptors are required for nociception. Mol. Cell Neurosci..

[18] Baur R., Beech R., Sigel E., Rufener L. (2014). Monepantel irreversibly binds to and opens *Haemonchus contortus* MPTL-1 and *Caenorhabditis elegans* ACR-20 receptors. Mol. Pharmacol..

[19] Kaminsky R., Ducray P., Jung M., Clover R., Rufener L., Bouvier J., Weber S.S., Wenger A., Wieland-Berghausen S., Goebel T. (2008). A new class of anthelmintics effective against drug-resistant nematodes. Nature.

[20] Karadzovska D., Seewald W., Browning A., Smal M., Bouvier J., Giraudel J.M. (2009). Pharmacokinetics of monepantel and its sulfone metabolite, monepantel sulfone, after intravenous and oral administration in sheep. J. Vet. Pharmacol. Ther..

[21] Rufener L., Baur R., Kaminsky R., Mäser P., Sigel E. (2010). Monepantel allosterically activates DEG-3/DES-2 channels of the gastrointestinal nematode *Haemonchus contortus*. Mol. Pharmacol..

[22] Rufener L., Bedoni N., Baur R., Rey S., Glauser D.A., Bouvier J., Beech R., Sigel E., Puoti A. (2013). acr-23 Encodes a monepantel-sensitive channel in *Caenorhabditis elegans*. PLoS Pathog..

[23] Bekdash R.A. (2019). Neuroprotective Effects of Choline and Other Methyl Donors. Nutrients.

[24] Rufener L., Keiser J., Kaminsky R., Mäser P., Nilsson D. (2010). Phylogenomics of Ligand-Gated Ion Channels Predicts Monepantel Effect. PLoS Pathog..

[25] Hansen T.V.A., Grencis R.K., Issouf M., Neveu C., Charvet C.L. (2021). Functional characterization of the oxantel-sensitive acetylcholine receptor from *Trichuris muris*. Pharmaceuticals.

[26] Boulin T., Gielen M., Richmond J.E., Williams D.C., Paoletti P., Bessereau J.-L. (2008). Eight genes are required for functional reconstitution of the *Caenorhabditis elegans* levamisole-sensitive acetylcholine receptor. Proc. Natl. Acad. Sci. USA.

